# Supplementary material for: A Likelihood Approach for Real-Time Calibration of Stochastic Compartmental Epidemic Models
Source: PLoS Comput Biol. 2017 Jan 17;13(1):e1005257. doi: 10.1371/journal.pcbi.1005257 (PMC5240920; doi:10.1371/journal.pcbi.1005257)
Supplement: S1 File — (TAR.GZ) [file pcbi.1005257.s014.tar.gz › HSPH_Online-SI-Revision/Additions/IPoi10/n100-extreme-SEITR_noisy/n100-extreme-SEITR_noisy_BENCH_table-4.pdf]

1	R0	{1.38654, 9.85565}	{2.7811, 19.6712}	{{2.7811, 19.6712}, {2.7811, 19.6712}}	{100.578, 99.5931}	inf	4942.	12 620.7	{2676., 23 192.}	97.6161	0.351247	newly	7087.2	5214.93	{1225., 9061.}	38.7149	0.297777	Reff	1.09645	2.15356	{1.55516, 2.66746}	96.7954	ar	92 379.	92 334.8	{92 148., 92 494.}	0.114698
2	R0	{2.36629, 12.2029}	{2.7943, 13.5962}	{{2.7943, 13.5962}, {2.7943, 13.5962}}	{18.0878, 11.4178}	inf	1894.	1162.65	{184., 2150.}	62.7167	0.579585	newly	3034.	951.111	{157., 1737.}	69.9828	0.69041	Reff	2.22479	2.74317	{2.70309, 2.78664}	23.3003	ar	99 022.	92 458.5	{92 306., 92 610.}	6.62834
3	R0	{2.32253, 12.836}	{2.92239, 11.5217}	{{2.85038, 10.7113}, {2.93597, 11.6547}}	{25.8277, 10.2394}	inf	1753.	1628.35	{205., 3364.}	63.5071	0.539526	newly	2684.	1757.1	{206., 3551.}	62.6221	0.5158	Reff	2.19238	2.8543	{2.73571, 2.9274}	30.1921	ar	98 997.	93 530.4	{93 315., 93 717.}	5.52194
4	R0	{1.79075, 11.2058}	{2.6266, 11.0527}	{{2.6266, 11.0527}, {2.6266, 11.0527}}	{46.676, 1.36626}	inf	2803.	5219.51	{597., 11 666.}	84.442	0.465568	newly	3933.	4847.22	{615., 10 074.}	76.7243	0.436759	Reff	1.61667	2.39342	{2.09668, 2.60031}	48.0461	ar	96 920.	89 393.2	{90 493., 90 947.}	7.76595
5	R0	{2.26952, 9.62371}	{2.00629, 6.32203}	{{1.90341, 5.70177}, {2.65613, 9.8939}}	{15.8349, 35.9062}	inf	1270.	461.326	{87., 1044.}	77.1673	0.828463	newly	3231.	928.391	{163., 2280.}	71.2661	0.744619	Reff	2.17415	2.01646	{1.86224, 2.65047}	14.1686	ar	98 881.	79 399.8	{76 351., 91 078.}	19.7017
6	R0	{1.57191, 6.89004}	{2.52921, 10.0481}	{{2.37615, 9.11994}, {2.9884, 12.8326}}	{60.9006, 45.8352}	inf	1343.	916.984	{66., 2038.}	58.6061	0.584975	newly	3690.	1117.16	{105., 2402.}	69.7248	0.750203	Reff	1.49482	2.49983	{2.29843, 2.9708}	67.2322	ar	95 046.	89 202.5	{87 307., 94 000.}	6.14812
7	R0	{1.99226, 17.2339}	{2.64334, 17.2462}	{{2.64334, 17.2462}, {2.64334, 17.2462}}	{32.6805, 0.071371}	inf	7484.	12 257.6	{3509., 21 469.}	39.5436	0.226067	newly	6848.	5867.9	{1970., 9545.}	33.089	0.201196	Reff	1.39466	2.04675	{1.53694, 2.49262}	48.2328	ar	97 992.	90 976.7	{90 844., 91 109.}	7.15903
8	R0	{2.63369, 15.1879}	{2.87181, 13.004}	{{2.87181, 13.004}, {2.87181, 13.004}}	{9.04131, 14.3792}	inf	3695.	1598.06	{176., 3759.}	73.3019	0.813949	newly	4729.	1396.26	{156., 3271.}	70.7164	0.763402	Reff	2.31741	2.80048	{2.70473, 2.86429}	20.8451	ar	99 451.	93 111.6	{92 960., 93 267.}	6.37436
9	R0	{2.53271, 9.90552}	{2.68374, 11.2101}	{{2.6266, 11.0527}, {2.80569, 11.6897}}	{6.87844, 14.1244}	inf	823.	261.529	{16., 592.}	79.2767	1.00786	newly	2227.	287.786	{19., 607.}	87.0774	1.22297	Reff	2.46668	2.67931	{2.60593, 2.80249}	9.42249	ar	99 355.	87 411.6	{88 891., 92 650.}	12.021
10	R0	{2.48791, 18.9003}	{2.99911, 15.6207}	{{2.99911, 15.6207}, {2.99911, 15.6207}}	{20.5474, 17.3521}	inf	5017.	14 981.6	{4560., 23 833.}	100.412	0.325197	newly	4646.	8218.33	{3022., 12 056.}	94.0046	0.302315	Reff	2.05964	2.23725	{1.74479, 2.78779}	17.7725	ar	99 291.	94 044.7	{93 904., 94 220.}	5.28373
11	R0	{2.84414, 18.4765}	{2.98125, 18.4749}	{{2.98125, 18.4749}, {2.98125, 18.4749}}	{4.82079, 0.00865965}	inf	3971.	4089.68	{830., 7083.}	51.6426	0.390438	newly	4035.	2144.27	{493., 3730.}	53.8802	0.430997	Reff	2.47881	2.7925	{2.64336, 2.94121}	13.1769	ar	99 654.	93 931.4	{93 806., 94 084.}	5.74245
12	R0	{2.29369, 14.622}	{2.86605, 14.6757}	{{2.86193, 14.6465}, {2.86193, 14.6465}}	{24.9535, 0.367433}	inf	4819.	4373.49	{629., 10 203.}	54.249	0.478782	newly	5911.	3016.75	{510., 6615.}	53.9573	0.480283	Reff	1.91186	2.66842	{2.39275, 2.84101}	39.572	ar	98 982.	93 060.1	{92 852., 93 232.}	5.98283
13	R0	{2.73413, 16.7529}	{2.7943, 13.5962}	{{2.7943, 13.5962}, {2.7943, 13.5962}}	{2.2007, 18.8427}	inf	4263.	5115.41	{863., 9965.}	47.7074	0.337619	newly	4789.	3788.2	{716., 7021.}	43.4825	0.300132	Reff	2.35852	2.56249	{2.34039, 2.7566}	10.1162	ar	99 543.	92 455.6	{92 277., 92 638.}	7.11995
14	R0	{1.79554, 16.5943}	{2.41197, 16.4137}	{{2.41197, 16.4137}, {2.41197, 16.4137}}	{34.3312, 1.08833}	inf	6015.	15 027.5	{3678., 21 889.}	71.3167	0.295816	newly	5421.	6963.24	{2010., 9409.}	50.61	0.242671	Reff	1.3756	1.61551	{1.07798, 2.25741}	28.203	ar	96 821.	88 025.4	{87 823., 88 248.}	9.08444
15	R0	{1.91429, 13.7771}	{2.7811, 19.6712}	{{2.7811, 19.6712}, {2.7811, 19.6712}}	{45.281, 42.7819}	inf	5411.	10 190.8	{2203., 19 483.}	64.2361	0.314115	newly	6031.	4328.95	{1079., 7845.}	43.0372	0.321506	Reff	1.53354	2.2888	{1.80746, 2.6869}	49.9207	ar	97 541.	92 299.5	{92 118., 92 453.}	5.37366
16	R0	{1.42094, 10.5871}	{2.58655, 14.3523}	{{2.58655, 14.3523}, {2.58655, 14.3523}}	{82.0309, 35.564}	inf	3733.	15 856.7	{2937., 24 124.}	198.179	0.471628	newly	4920.	8617.95	{2063., 11 834.}	95.226	0.322085	Reff	1.20628	1.7499	{1.1282, 2.46027}	47.9062	ar	93 044.	90 301.1	{90 021., 90 582.}	2.94792
17	R0	{1.45515, 6.16466}	{2.72175, 10.8305}	{{2.68409, 10.6354}, {2.91946, 11.8546}}	{87.0425, 75.6864}	inf	733.	472.921	{38., 1096.}	63.8726	0.686785	newly	2188.	551.206	{43., 1280.}	74.8078	0.939296	Reff	1.41733	2.70493	{2.64053, 2.90369}	90.847	ar	93 254.	90 292.3	{91 196., 93 492.}	3.26068
18	R0	{2.85669, 13.2416}	{2.67672, 12.1364}	{{2.52844, 11.113}, {2.95209, 14.037}}	{8.63771, 12.5512}	inf	1540.	636.426	{88., 1466.}	74.5676	0.865864	newly	2807.	603.426	{73., 1471.}	78.5028	0.937615	Reff	2.71777	2.647	{2.47668, 2.94406}	7.7802	ar	99 644.	91 016.4	{89 452., 93 743.}	8.65838
19	R0	{2.56417, 17.9558}	{2.97157, 19.3547}	{{2.97157, 19.3547}, {2.97157, 19.3547}}	{15.8882, 7.7908}	inf	4153.	3224.	{179., 8440.}	63.1195	0.651798	newly	4054.	1586.99	{99., 4021.}	63.8715	0.698127	Reff	2.21442	2.82361	{2.57917, 2.96289}	27.5101	ar	99 389.	93 865.7	{93 747., 94 047.}	5.55721
20	R0	{2.27517, 11.1864}	{2.81598, 11.7741}	{{2.80569, 11.6897}, {2.9884, 12.8326}}	{23.7701, 5.45283}	inf	2051.	913.187	{107., 2207.}	71.1034	0.78243	newly	3692.	935.625	{107., 2264.}	74.658	0.849275	Reff	2.12451	2.76747	{2.64661, 2.80549}	30.2641	ar	98 894.	92 530.	{92 252., 92 872.}	6.43517
21	R0	{2.35735, 10.4735}	{2.75567, 11.0134}	{{2.68409, 10.6354}, {2.91946, 11.8546}}	{16.897, 5.15448}	inf	1087.	322.077	{37., 740.}	80.02	0.924771	newly	2417.	365.831	{39., 823.}	84.8643	1.04101	Reff	2.2751	2.75048	{2.66525, 2.91581}	20.8947	ar	99 036.	92 122.5	{91 251., 93 617.}	6.98077
22	R0	{1.79551, 11.9179}	{2.81617, 13.9425}	{{2.81617, 13.9425}, {2.81617, 13.9425}}	{56.8451, 16.9879}	inf	4439.	16 180.6	{4430., 25 985.}	146.869	0.384746	newly	5958.	9527.79	{3408., 13 623.}	78.1761	0.269491	Reff	1.5037	1.97472	{1.31025, 2.62011}	36.6072	ar	97 033.	92 619.6	{92 443., 92 825.}	4.54835
23	R0	{1.23545, 6.31687}	{2.7628, 12.2542}	{{2.47628, 10.2848}, {2.87181, 13.004}}	{123.627, 93.9919}	inf	2814.	4387.48	{878., 9210.}	60.8366	0.326489	newly	6598.	3828.13	{736., 7255.}	47.9476	0.402517	Reff	1.0892	2.56011	{2.2919, 2.82684}	135.045	ar	88 739.	91 896.8	{88 873., 93 236.}	3.55851
24	R0	{2.24089, 10.1789}	{2.7943, 13.5962}	{{2.7943, 13.5962}, {2.7943, 13.5962}}	{24.696, 33.5724}	inf	976.	419.219	{26., 921.}	73.8144	0.847519	newly	2027.	350.672	{25., 797.}	82.7	1.06679	Reff	2.17337	2.77605	{2.75403, 2.7931}	27.7301	ar	98 868.	92 466.	{92 297., 92 625.}	6.47528
25	R0	{1.57298, 10.2607}	{2.58655, 14.3523}	{{2.58655, 14.3523}, {2.58655, 14.3523}}	{64.4363, 39.8764}	inf	2641.	3640.82	{554., 7973.}	62.366	0.414556	newly	3941.	2451.15	{415., 5268.}	53.2004	0.458832	Reff	1.41523	2.42675	{2.23905, 2.56296}	71.4741	ar	94 957.	90 345.5	{90 101., 90 567.}	4.85637
26	R0	{1.2154, 8.2576}	{2.8833, 17.1847}	{{2.8833, 17.1847}, {2.8833, 17.1847}}	{137.231, 108.108}	inf	4010.	9247.25	{816., 18 688.}	104.261	0.455584	newly	6408.	4715.01	{476., 8984.}	48.0349	0.429437	Reff	0.993966	2.43861	{1.96111, 2.84677}	145.342	ar	88 421.	93 159.3	{93 013., 93 310.}	5.35874
27	R0	{2.22841, 18.7983}	{2.52651, 14.1724}	{{2.52651, 14.1724}, {2.52651, 14.1724}}	{13.3773, 24.6081}	inf	7582.	15 971.4	{2410., 23 461.}	55.7601	0.300756	newly	6511.	8498.46	{1642., 11 646.}	58.682	0.276831	Reff	1.58429	1.62	{0.866921, 2.42459}	29.3145	ar	98 756.	89 605.6	{89 372., 89 813.}	9.26571
28	R0	{1.27778, 5.36767}	{2.46369, 8.47708}	{{2.21381, 7.10188}, {2.74924, 10.0386}}	{92.8103, 57.9284}	inf	966.	2061.02	{271., 3912.}	103.922	0.421789	newly	2971.	3055.7	{424., 5742.}	65.1654	0.40407	Reff	1.23118	2.37814	{2.07226, 2.72573}	93.1596	ar	89 843.	88 107.9	{84 439., 92 326.}	4.28294
29	R0	{2.38042, 15.8901}	{2.81617, 13.9425}	{{2.81617, 13.9425}, {2.81617, 13.9425}}	{18.3056, 12.2567}	inf	3337.	9656.8	{1943., 16 922.}	115.186	0.416177	newly	3642.	6365.15	{1524., 10 571.}	102.851	0.388766	Reff	2.11469	2.35832	{1.98858, 2.73011}	15.5901	ar	99 112.	92 657.3	{92 508., 92 780.}	6.51249
30	R0	{2.53188, 12.7607}	{2.80098, 11.6736}	{{2.80569, 11.6897}, {2.80569, 11.6897}}	{10.6284, 8.5307}	inf	1016.	338.	{47., 713.}	76.6721	0.861012	newly	1649.	362.375	{47., 806.}	78.1326	0.898168	Reff	2.45506	2.78598	{2.77211, 2.8035}	13.4788	ar	99 317.	92 496.4	{92 349., 92 716.}	6.86747
31	R0	{1.24482, 10.5471}	{2.65223, 17.3283}	{{2.64334, 17.2462}, {2.64334, 17.2462}}	{113.062, 64.2941}	inf	5483.	17 577.8	{8615., 25 025.}	112.826	0.312881	newly	6713.	7806.06	{4370., 10 163.}	34.3979	0.145829	Reff	0.914831	1.68763	{1.05839, 2.28371}	85.8397	ar	89 097.	91 010.1	{90 743., 91 202.}	2.14724
32	R0	{2.30446, 17.9607}	{2.7943, 13.5962}	{{2.7943, 13.5962}, {2.7943, 13.5962}}	{21.2562, 24.3003}	inf	5816.	9074.2	{2176., 18 376.}	54.8568	0.363094	newly	5244.	6136.72	{1753., 11 562.}	61.1272	0.325595	Reff	1.82382	2.36228	{1.89632, 2.69711}	30.7304	ar	99 028.	92 445.7	{92 288., 92 601.}	6.64689
33	R0	{2.31579, 11.8236}	{2.77472, 9.36216}	{{2.37035, 7.13655}, {2.9194, 10.1535}}	{19.8173, 20.8181}	inf	3092.	9350.64	{1814., 19 939.}	126.894	0.363115	newly	5187.	10 197.2	{2670., 17 641.}	118.836	0.343607	Reff	2.08296	2.3412	{1.78177, 2.82283}	20.3055	ar	99 012.	92 049.8	{87 389., 93 564.}	7.03169
34	R0	{1.6678, 10.6744}	{2.7943, 13.5962}	{{2.7943, 13.5962}, {2.7943, 13.5962}}	{67.5441, 27.372}	inf	4713.	8986.	{1394., 17 183.}	74.5287	0.353659	newly	6918.	6120.51	{1153., 10 936.}	47.9669	0.318168	Reff	1.37288	2.36983	{1.96314, 2.73162}	72.6169	ar	95 809.	92 454.8	{92 308., 92 644.}	3.50098
35	R0	{1.65476, 7.07559}	{2.69821, 10.7086}	{{2.68409, 10.6354}, {2.68409, 10.6354}}	{63.0576, 51.345}	inf	893.	377.857	{18., 766.}	72.7366	0.838993	newly	2514.	449.054	{24., 945.}	82.1379	1.05492	Reff	1.60409	2.68459	{2.65059, 2.6835}	67.359	ar	95 746.	88 235.1	{91 176., 91 635.}	7.84456
36	R0	{2.71807, 18.5959}	{2.58655, 14.3523}	{{2.58655, 14.3523}, {2.58655, 14.3523}}	{4.83873, 22.8201}	inf	3397.	1514.42	{92., 3612.}	71.426	0.804096	newly	3163.	1066.39	{82., 2486.}	66.3994	0.740147	Reff	2.4286	2.52172	{2.43244, 2.58236}	4.03554	ar	99 540.	90 317.2	{90 115., 90 507.}	9.26539
37	R0	{2.6202, 12.7552}	{2.5065, 14.0053}	{{2.48103, 13.7658}, {2.48103, 13.7658}}	{5.71747, 9.80087}	inf	1248.	598.833	{47., 1362.}	69.2253	0.716304	newly	2178.	443.583	{30., 988.}	79.6335	0.923346	Reff	2.51914	2.49007	{2.42412, 2.48004}	3.52019	ar	99 426.	89 368.1	{88 823., 89 377.}	10.1159
38	R0	{1.47672, 9.81651}	{2.58655, 14.3523}	{{2.58655, 14.3523}, {2.58655, 14.3523}}	{75.1551, 46.2057}	inf	4210.	10 597.6	{2545., 19 478.}	91.5235	0.361345	newly	6266.	6305.81	{1796., 10 697.}	44.6624	0.291354	Reff	1.23	2.08087	{1.59329, 2.47923}	69.225	ar	93 815.	90 340.5	{90 174., 90 569.}	3.7036
39	R0	{1.90004, 12.3313}	{2.99911, 15.6207}	{{2.99911, 15.6207}, {2.99911, 15.6207}}	{57.8446, 26.6752}	inf	3615.	8440.62	{1403., 17 621.}	89.4979	0.36851	newly	4980.	5088.77	{977., 9804.}	52.726	0.32451	Reff	1.65284	2.59346	{2.12058, 2.93799}	56.909	ar	97 413.	94 040.9	{93 886., 94 185.}	3.46168
40	R0	{2.64218, 18.5237}	{2.86895, 12.986}	{{2.85273, 12.8842}, {2.87181, 13.004}}	{8.58261, 29.8951}	inf	6412.	15 327.7	{3697., 26 230.}	74.923	0.302745	newly	6088.	9970.3	{3163., 14 871.}	86.5932	0.296295	Reff	2.0572	2.07564	{1.42827, 2.70435}	20.0794	ar	99 474.	93 081.6	{92 924., 93 242.}	6.42618
41	R0	{1.6945, 7.17996}	{2.85381, 13.4925}	{{2.86001, 13.5542}, {2.86001, 13.5542}}	{68.4161, 87.9189}	inf	498.	155.045	{16., 394.}	76.2201	0.909103	newly	1333.	132.955	{12., 350.}	90.0259	1.28698	Reff	1.66739	2.85299	{2.84268, 2.85909}	71.1052	ar	96 332.	91 606.1	{92 815., 93 168.}	4.90589
42	R0	{1.84559, 18.0074}	{2.64334, 17.2462}	{{2.64334, 17.2462}, {2.64334, 17.2462}}	{43.2247, 4.22715}	inf	7319.	14 824.	{4751., 23 993.}	48.3164	0.27766	newly	6151.	6791.8	{2563., 10 124.}	42.4291	0.247692	Reff	1.29117	1.89118	{1.28847, 2.43597}	47.9749	ar	97 358.	90 993.9	{90 841., 91 178.}	6.5368
43	R0	{2.24965, 17.0361}	{2.61082, 12.9924}	{{2.44675, 11.6528}, {2.7346, 14.003}}	{16.0547, 23.736}	inf	4414.	12 144.1	{1606., 23 427.}	104.473	0.402066	newly	4281.	7719.41	{1422., 12 976.}	107.399	0.385368	Reff	1.90728	2.02852	{1.35521, 2.57856}	20.2603	ar	98 770.	90 710.5	{88 382., 92 059.}	8.15985
44	R0	{2.54324, 15.7967}	{2.7346, 14.003}	{{2.7346, 14.003}, {2.7346, 14.003}}	{7.52426, 11.3549}	inf	4842.	3631.15	{329., 7776.}	61.4873	0.62976	newly	5449.	2600.82	{244., 5354.}	59.0069	0.600529	Reff	2.13836	2.5721	{2.38774, 2.72084}	20.3394	ar	99 354.	91 879.3	{91 681., 92 050.}	7.52333
45	R0	{2.54943, 14.8032}	{2.87181, 13.004}	{{2.87181, 13.004}, {2.87181, 13.004}}	{12.6452, 12.1541}	inf	3565.	1472.88	{113., 3709.}	74.2913	0.887699	newly	4552.	1288.46	{104., 3111.}	72.181	0.841717	Reff	2.25387	2.80637	{2.70944, 2.86661}	24.5131	ar	99 380.	93 090.6	{92 934., 93 263.}	6.32863
46	R0	{2.37755, 12.6461}	{2.93597, 11.6547}	{{2.93597, 11.6547}, {2.93597, 11.6547}}	{23.4872, 7.83957}	inf	1629.	4380.66	{521., 8619.}	105.778	0.36409	newly	2717.	4242.26	{603., 8068.}	91.5316	0.338137	Reff	2.25026	2.73455	{2.50033, 2.90858}	21.675	ar	99 096.	93 587.2	{93 396., 93 754.}	5.55903
47	R0	{1.69279, 11.6636}	{2.5684, 13.7324}	{{2.5684, 13.7324}, {2.5684, 13.7324}}	{51.7258, 17.7372}	inf	3683.	7655.1	{1058., 14 159.}	77.6662	0.337376	newly	4864.	5052.75	{842., 8841.}	52.8163	0.298872	Reff	1.4668	2.22014	{1.90999, 2.52445}	51.3591	ar	96 214.	90 115.1	{89 896., 90 307.}	6.33894
48	R0	{1.82402, 10.0038}	{2.93091, 13.8908}	{{2.95209, 14.037}, {2.95209, 14.037}}	{60.684, 38.8552}	inf	1751.	3724.94	{287., 8179.}	99.8301	0.433717	newly	3183.	2850.18	{251., 6168.}	64.3637	0.436078	Reff	1.71321	2.76853	{2.56212, 2.93907}	61.5989	ar	97 076.	93 572.8	{93 504., 93 853.}	3.60876
49	R0	{1.6424, 11.1667}	{2.95209, 14.037}	{{2.95209, 14.037}, {2.95209, 14.037}}	{79.7425, 25.7041}	inf	5534.	21 994.3	{10 732., 29 260.}	155.6	0.40906	newly	7611.	12 187.2	{7497., 14 517.}	68.8929	0.243183	Reff	1.28365	1.72375	{1.12215, 2.44528}	40.1098	ar	95 546.	93 704.7	{93 577., 93 855.}	1.92715
50	R0	{2.05638, 14.3048}	{2.95209, 14.037}	{{2.95209, 14.037}, {2.95209, 14.037}}	{43.5576, 1.8721}	inf	4454.	6819.97	{1606., 14 740.}	55.0494	0.313751	newly	5024.	4859.96	{1308., 9617.}	49.1477	0.285877	Reff	1.73898	2.63174	{2.24291, 2.87965}	51.3385	ar	98 194.	93 731.9	{93 595., 93 883.}	4.54413
